# Body image disturbances, fear and associations with the amygdala in anorexia nervosa

**DOI:** 10.1007/s00508-018-1440-y

**Published:** 2019-01-17

**Authors:** Nathalie T. Burkert, Karl Koschutnig, Franz Ebner, Wolfgang Freidl

**Affiliations:** 1 Institute of Social Medicine and Epidemiology, Medical University Graz, Universitaetsplatz 6/I, 8010 Graz, Austria; 2 Institute of Psychology, Karl Franzens University Graz, Graz, Austria; 3 Clinic of Neuroradiology, General Hospital Graz, Graz, Austria

**Keywords:** Amygdala, Comorbidities, Body image, MRI measurement, Anorexia nervosa

## Abstract

**Background:**

Anorexia nervosa (AN) is a severe illness with a high mortality rate which mainly affects young women. Studies found a localized volume loss of the amygdala in patients with AN, a brain region responsible for affective responses. Patients with AN were found to have body image distortions, and suffer from the comorbid disorders depression, anxiety disorder, and obsession. Therefore, the purpose of this study was to analyze a possible connection between comorbidities, body image disturbances, and the volume of the amygdala in patients with AN.

**Methods:**

In this study 21 females suffering from restrictive-type AN and 21 age-matched normal controls (NC) were tested. Demographic data as well as body image perceptions and comorbidities were assessed. Volumes of cortical structures were measured with a magnetic resonance (MR) scanner. Analyses of variance were conducted to analyze group differences, and correlations between the volume of the amygdala and comorbidities and body image perceptions were calculated.

**Results:**

The results showed a significantly lower grey matter volume in the amygdala in AN patients compared to the NC. Persons with AN showed more body image disturbances and suffered more often from depression, and phobias than NC. The volume of the amygdala showed a non-significant mid-level association with phobia and with uncertainty concerning their body in AN patients.

**Conclusion:**

The study indicates that phobic anxiety and body image in patients with AN could be related to the volume of the amygdala. The results contribute to a better understanding of the pathophysiology of the disease.

## Introduction

Anorexia nervosa (AN) is one of the most severe mental illnesses, which mainly affects young women [1, 2]. The disease is characterized by a persistent desire to stay extremely thin, a pathological fear of weight gain combined with a distortion of one’s own body perception. These symptoms are accompanied by specific personality characteristics such as harm avoidance, perfectionism, obsessive behavior, emotionality and social insecurity [3, 4]. Additionally, AN is related to severe medical complications, nutritional and endocrine changes [5] as well as structural [4, 6–8] and functional brain alterations [4, 6, 7].

Functional alterations in the ventral limbic system, i.e.amygdala, insula, striatum, anterior cingulate cortex (ACC) and orbitofrontal cortex, concerning the processing of emotional stimuli seem to exist in patients with AN (even after recovery) which might be central regarding development and maintenance of the disease [9, 10]. The limbic structures which are the neural basics of emotions include the amygdala, hippocampus, cingulate cortex and olfactory cortex [11]. Previous studies showed that patients with AN have a reduced grey matter volume in several brain regions, including subcortical regions like the amygdala or putamen [12–14]. Giordano et al. [15] found a significant reduction of the hippocampus-amygdala formation in patients with AN, even after recovery. These disturbances in neurobiological systems have been implicated to influence diverse psychopathological symptoms of the disease [12]. A hyperactivation of the amygdala was observed concerning negative feedback or aversive stimuli suggesting an elevated negative arousal in AN [16–18]. Moreover, evidence suggests that an increased brain activity in the amygdala (as well as the anterior cingulate cortex (ACC) and prefrontal regions) might be involved in fearful emotional processing concerning body images and might in turn influence weight gain [19–23].

Only a limited number of publications focused on the role of the volume of the amygdala concerning certain symptoms in AN [12–15]. Therefore, the purpose of this study was to compare body image perceptions, comorbidities such as phobia or depression, and the volume of the amygdala, as well as possible associations between structural changes of the amygdala with psychological symptoms in patients with AN and in normal eating individuals.

## Material and methods

### Participants

A total of 21 females currently affected by restrictive type AN and 21 age-matched normal controls (NC) were recruited in treatment centers and through advertisements. The patients with AN had been diagnosed with the illness for more than 1 year. They were suffering from restrictive type AN and had a body mass index (BMI) below 17.5 kg/m^2^. Restrictive eating patterns were defined as regular restrictive food intake, avoiding high-caloric food, counting calories, and dieting. We excluded females who reported binge eating or compensatory behavior to reduce weight, such as vomiting or laxative abuse in their previous history. Moreover, the participants were not using any illicit drugs or abusing alcohol. The NC had a BMI in the normal range (between 18.5 and 25 kg/m^2^) and no psychiatric illness or eating disorder in their previous medical history. They did not have any first-degree relative with a psychiatric disorder and were not taking any medication, except for birth control.

The study was carried out in compliance with the Declaration of Helsinki. Written informed consent was obtained from all participants or from their parents if they were under age. The ethics committee of the Medical University of Graz approved this study (EK-number: 23-217 ex 10/11).

### Test procedure

First, a clinical psychologist interviewed all participants with the structural clinical interview for DSM-IV axis I disorders [24] to assess the eating disorder and potential comorbid axis I psychopathology, as well as inclusion and exclusion criteria. Additionally, all participants completed the eating disorder inventory (EDI-2) [25] to assess their eating behavior. Participants who passed the first screening underwent an extended examination. Their weight and height were measured and sociodemographic and medical data were collected. Furthermore, information regarding their body image (questionnaire concerning attitudes toward ones own body, FBeK) [26] was gathered. The questionnaire measured the subjective evaluation of a person’s own body concerning the following four dimensions: attractivity/confidence, accentuation of body appearance, uncertainty/concerned, physical-sexual discomfort. Moreover, potential comorbidities (phobia, depression, and obsession) were measured with the symptom checklist (SCL-90R) [27]. The SCL-90R captures the subjective impairment due to physical and psychological symptoms in nine dimensions on a 5-point Likert scale. On another day the structural brain measurement using magnetic resonance imaging (MRI) was conducted which took approximately 15 min.

This study also included measurement of other psychological aspects of the disease (i. e. personality, stress, coping) as well as taste processing due to the administration of three different tastes. Some results have been published previously (e. g. the volume of hippocampal substructures and their associations with stress and coping). For further details please refer to Burkert (2016) [4] and Burkert et al. (2015) [8].

### Scanning procedure (MRI measurement)

Imaging was performed on a 3.0 T Tim Trio system (Siemens Medical Systems, Erlangen, Germany) using a 12-channel head coil. High-resolution anatomical images were obtained using a T1-weighted 3D MPRAGE sequence (TR = 1900 ms, TE = 2.2 ms, 176 sagittal slices) which provided 1 × 1 × 1 mm isotropic resolution.

### Image analyses

The amygdala volume was obtained using the FreeSurfer software package (Martinos Center for Biomedical Imaging, Boston, MA, USA) by applying the automated whole brain segmentation procedure to label each voxel in native space for each participant [28]. In brief, this process includes removal of nonbrain tissue, automated transformation to Talairach space, and segmentation of subcortical grey and white matter structures (including the amygdala).

### Statistical analyses

Between group differences concerning body image and potential comorbidities such as phobia, depression and obsession, as well as regarding the volume of the amygdala were analyzed with multivariate analyses of variance (MANOVA) to minimize multiple comparisons. Differences between the groups in single variables or scales were calculated by analyses of variance.

In a second step, correlations were calculated between the overall volume of the left and right amygdalae with body image subscales and comorbidities using Pearson’s correlations. The strength of the association was interpreted as follows: correlations higher than r = 0.3 and lower or equal to r = 0.5 were interpreted as mid-level associations and correlations higher than r = 0.5 as high-level associations [29].

All analyses were calculated using IBM SPSS software (Version 22.0 for windows; IBM Austria, Vienna). A statistical threshold of *p* < 0.05 was set to reject the null hypothesis.

## Results

### Behavioral results

Patients with AN and the normal controls were of similar age (AN: M = 21.6 years, SD = 5.7 years; NC: M = 21.7 years, SD = 5.7 years, F_1,40_ = 0.002, *p* = 0.968). The mean age at the onset of the disease was 15.6 years (SD = 3.2 years), and the mean duration was 4.4 years (SD = 3.3 <ears). Of the patients with AN 7 (33%) were taking a selective serotonin reuptake inhibitor (SSRI).

### Body image and comorbidities

The results of the multivariate analyses of variance showed that individuals suffering from AN differed significantly from NC concerning body perceptions. They had lower values concerning their perception to be attractive, were more uncertain and concerned, and felt more physical-sexual discomfort (Table 1).

**Table 1:** Body Image and Comorbidities of AN vs. NC

	AN		NC		MANOVA	
	Mean	SD	Mean	SD	F	*p-*value
*Body perception (FBeK)*						
–	–	–	–	–	3.680	*0.013*
Attractivity/confidence	6.90	3.21	9.71	3.05	8.455	*0.006*
Accentuation of body appearance	7.95	1.56	8.62	1.60	1.868	0.179
Uncertainty/concerned	6.57	2.56	5.10	1.92	4.466	*0.041*
Physical-sexual discomfort	2.86	1.35	2.05	1.02	4.785	*0.035*
*Comorbidities (SCL-90R)*						
–	–	–	–	–	5.253	*0.004*
Depression	1.15	0.74	0.47	0.53	11.648	*0.001*
Phobia	0.46	0.66	0.12	0.26	4.777	*0.035*
Obsession	0.97	0.73	0.57	0.59	3.785	0.059

Group comparison by analyses of variance. The significant levels are in italics

*AN* women with anorexia nervosa, restrictive type, *NC* normal control eaters, *SD* standard deviation, *MANOVA* multivariate analyses of variance, *FBeK* questionnaire concerning attitudes toward ones own body, *SCL-90R* symptom checklist

The AN group suffered from significantly more comorbidities than NC. The analyses of variance showed that AN had a higher score concerning depression and phobia, but not regarding obsession compared to NC (Table 1).

### Brain volume and volume of the amygdala

The two groups did not differ in their total intracranial volume (AN: M = 1408.2 cm^3^, SD = 167.2; NC: M = 1480.0 cm^3^, SD = 95.0; F_1,36_ = 0.8, *p* = 0.173) or cortical white matter volume (AN: M = 455.4 cm^3^, SD = 66.0; NC: M = 471.3 cm^3^, SD = 54.3; F_1,36_ = 0.7, *p* = 0.712). Analysis of variance showed that AN have significantly less grey matter volume (AN: M = 647.0 cm^3^, SD = 56.8; NC: M = 709.4 cm^3^, SD = 64.3; F_1,36_ = 5.5, *p* = 0.004) compared to NC. Moreover, AN had a significantly smaller left and right amygdala compared to NC (right amygdala: AN: M = 1433.7 mm^3^, SD = 176.8; NC: 1580.7 mm^3^, SD = 144.2; F F_1,40_ = 8.725, *p* = 0.005, left amygdala: AN: M = 1397.8 mm^3^, SD = 217.1; NC: M = 1579.9 mm^3^, SD = 187.3; F_1,40_ = 8.474, *p* = 0.006).

### Associations between the volume of the amygdala with body image and comorbidities

A significant mid-level association was found between the volume of the amygdala and the total grey matter volume in patients with AN (r = 0.49, *p* = 0.023). Moreover, a significant high-level association was found between the volume of the amygdala and the cortical white matter volume in this group (r = 0.63, *p* = 0.002). A smaller volume of the amygdala is related to smaller white and grey matter volumes in AN. A mid-level non-significant association was found between the volume of the amygdala and the intracranial volume in AN (r = 0.36, *p* = 0.111). No association was found between the volume of the amygdala and the total grey matter volume (r = 0.20, *p* = 0.392), the cortical white matter volume (r = 0.19, *p* = 0.402), or the intracranial volume (r = 0.14, *p* = 0.546) in NC. No association existed between the duration of the illness with the volume of the amygdala in AN (r = −0.07, *p* = 0.774).

**Fig. 1:**
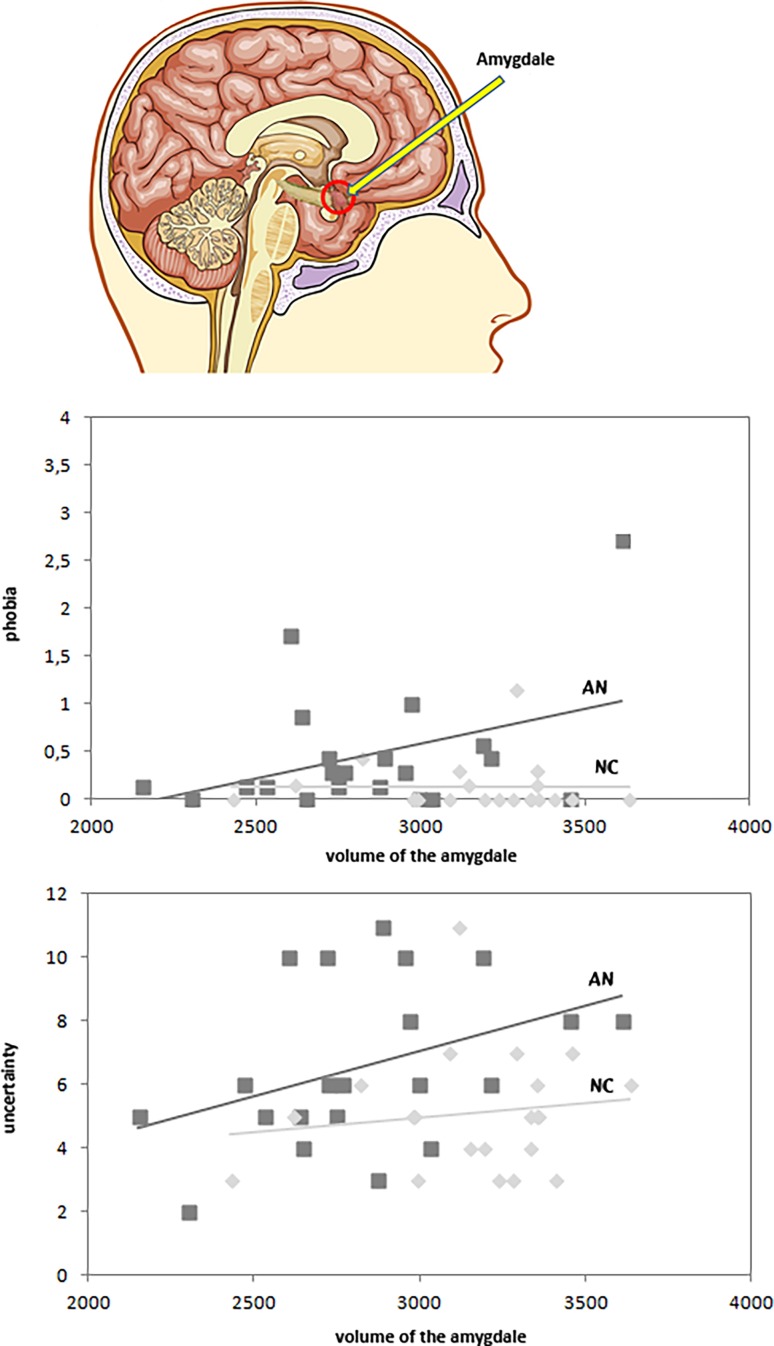
Association between the volume of the amygdala with phobia and uncertainty. Volume (in mm^3^) of the amygdala and its associations with phobia (using a scale from 0 not at all to 4 very much) and uncertainty concerning body and attractiveness (using a scale from 0 not at all to 12 high uncertainty) in AN vs. NC; *AN* women with anorexia nervosa, restrictive type, *NC* healthy control women. *N* = 42

The analyses revealed a mid-level association between the total volume of the amygdala and phobia in AN showing that smaller volumes related to less phobic anxiety (r = 0.39, *p* = 0.082, Fig. 1); however, this association was not significant. The results also showed a non-significant mid-level association between uncertainty concerning their body and the volume of the amygdala in patients with AN (r = 0.39, *p* = 0.078). Smaller volumes of the amygdala were related to less uncertainty. No associations were found between the volume of the amygdala with phobic anxiety (r = −0.01, *p* = 0.974) or uncertainty (r = 0.14, *p* = 0.556) in NC.

No association was found concerning depression or obsession in either AN or NC (depression: AN: r = 0.09, *p* = 0.688 NC: r = 0.03, *p* = 0.898; obsession: AN: r = 0.24, *p* = 0.301, NC: r = 0.17, *p* = 0.458). Moreover, no association was found between the volume of the amygdala and any other body image aspect in AN or NC (attractivity/confidence: AN: r = −0.04, *p* = 0.869, NC: r = 0.29, *p* = 0.196; accentuation of body appearance: AN: r = −0.05, *p* = 0.837, NC: r = 0.12, *p* = 0.603; physical sexual discomfort: AN: r = 0.12, *p* = 0.616, NC: r = −0.19, *p* = 0.413).

## Discussion

This study analysed associations between structural abnormalities of the amygdala with body image and comorbidities in patients suffering from AN compared to NC. The study provides evidence that individuals suffering from AN have a smaller volume of the amygdala bilaterally compared to NC. This is in line with previous studies which showed that patients with AN have a reduced localized grey matter volume concerning the amygdala [12–15].

Evidence exists that disturbances in neurobiological systems have implications concerning various psychopathological symptoms which accompany the disease [12]. In patients with AN impaired ability to process emotional stimuli was observed which persists even after recovery [9]. A ventral neurocircuit, which includes the amygdala is responsible for identifying the emotional aspects of stimuli and generates an affective response [11, 30]. Moreover, cellular and molecular plasticity in amygdala as well as hippocampal circuits mediates the reaction to fearful situations and related anxiety [31]. A greater activation in the amygdala was observed in functional MRI studies which indicates that emotional processing concerning bodily issues is disturbed in patients with AN [19–22]. Disturbances concerning body image in patients with AN are linked to changes in the prefrontal cortex, the amygdala and the insula [20]. Miyake et al. [21] suggested that an increased brain activity in the amygdala might lead to fearful emotional processing concerning body image issues, and in turn influences caloric intake and weight gain in patients with AN. In the present study, a reduction of the volume of the amygdala showed a mid-level association with less uncertainty concerning bodily issues as well as lower phobia scores in patients with AN; however, this association was not significant. No such association was found between the volume of the amygdala with the duration of illness, depression and obsession scores, or other body image issues in AN or NC in this study. It might be possible that the reduction of volume of the amygdala might be involved in reducing fear in patients with AN and lead to less body image uncertainty. As a consequence, this might contribute to maintenance of the disease. Since the study only analyzed data of 21 AN currently affected by the disease it would be interesting whether in further studies with more participants a significant association between a selective volume loss of the amygdala with fear and body image disturbances in AN can be found.

Scaife et al. [17] reported that viewing pictures of food in general is associated with decreased activity in somatosensory regions; however, since no brain activity as a response to the viewing of body pictures and associated fear ratings were measured in this study, further studies are needed to analyze this relationship in more detail. The results also showed that the volume of the amygdala is associated with the overall brain volume. Regarding volume reduction and functional alterations of the amygdala, it must be considered that this function is highly connected to other regions such as prefrontal brain areas or the insula [10, 30]. Results of a functional study in the patients which were analyzed in this study showed alterations in taste processing in the insula, ACC and frontal cortex. The brain activity in these areas was associated with stress and anxiety in patients [4]. Therefore, anxiety might also be linked to alterations of volume in other brain areas. This needs to be analyzed in further studies. The cross-sectional design of this study did not allow determination of whether the findings are traits that contribute to the onset of AN, or a consequence of malnutrition and weight loss; however, the reduced volume of the amygdala and its association with phobia and uncertainty of body image suggests that it could be possible that disturbances in emotional processing might at least in part be explained by neurobiological changes in AN.

In AN alterations concerning the functional connectivity between frontal brain regions and the ACC with the amygdala were observed [16, 32] indicating impairment of emotion regulation leading to higher anxiety [33]. The elevated activity in frontal brain regions (PFC) and the ACC seems to indicate that bottom up processes such as emotion processing in AN are associated with an increase in control activity [16–18, 32, 34]. The elevation suggests an effort is made in AN to compensate for the deficiencies in emotion regulation [33]. Moreover, a hypoconnectivity between the left amygdala with the dorsal striatum, the dorsal ACC and the medial PFC in AN is associated with greater subjective fear ratings, anxiety, severity of symptoms as well as obsessions and compulsions [17, 33]. The results showed that structural changes in the amygdala are related to fear and uncertainty. Whether other brain structures (such as the PFC or the ACC) are also related to emotions should further be analyzed. In an animal model of anorexia Aoki et al. [35] found an enhanced activity of the ventral hippocampus which was associated with anxiety regulation and led to an increase in amygdala activation. Therefore, in AN a higher anxiety seems to be present due to neurobiological changes in the hippocampus and associated regions [35]. In the present study, the patients with AN also had a reduction of the hippocampal volume which was associated with stress [8]. Whether other brain structures (such as the prefrontal cortex (PFC) or the ACC) are also related to emotions should further be analyzed.

### Strengths and limitations of the study

One of the strengths of this study are the matching of AN patients with normal controls based on age. Moreover, a relatively large sample of individuals with MRI measurement were tested; however, although on one hand testing 21 individuals in one MRI study is a relatively large sample, it is a relatively small sample size considering the fact that to obtain statistically significant results the larger the sample is, the more likely existing differences and associations can be found. Further prospective and longitudinal studies in a larger number of individuals should therefore be conducted to analyze neurobiological changes and associations with symptoms in patients with AN in more detail. A potential limitation of this study is that the brain volumes of participants currently affected by AN were analyzed. As a consequence, it was not possible to differentiate between the primary disorder and secondary phenomena that accompany the disease. Since malnutrition is associated with many changes concerning neuroendocrine function, cause and consequence remain unclear. The cross-sectional design of this study did not allow a differentiation as to whether the findings are traits that contribute to the onset of AN, or a consequence of malnutrition and weight loss. The results of previous studies have shown that it is still unknown whether structural and functional abnormalities are fully reversible. Some studies show an increase in brain volume after weight restoration in patients with AN [13, 36, 37], while others showed that structural changes of the brain persist after recovery [13, 15, 38]. It is also possible that only parts of the brain recover [14]. Monzon et al. [14] found that with weight recovery the majority of grey matter volumes in the brain recovers in patients with AN. It might be possible that recovery in grey matter volume also leads to recovery of psychological functions such as anxiety reduction or body shape concerns. Recovery of grey matter in the orbifrontal cortex (OFC) in AN was associated with less body shape concerns [14]. Whether such an association also exists regarding recovery of the amygdala and anxiety reduction should be analyzed in further studies. Moreover, further longitudinal studies are needed to analyze structural changes and associations with psychopathological symptoms in more detail.

## Conclusion

The results of this study suggest that it could be possible that disturbances in emotional processing might at least in part be explained by neurobiological changes in AN. To our knowledge, this is the first study indicating that the size of the amygdala might be associated with phobic anxiety and body uncertainty in patients with AN. The AN is the most severe psychiatric disorder which is associated with many medical complications [39], and there still is a lack of proven therapies for eating disorders [40]. Body image distortions and the fear in patients with AN contribute to the maintenance of the disease and lead to further dieting, which reduces anxiety but aggravates the symptoms of the disease [41]. Therefore, a better understanding of the pathophysiology of the disease, neurobiological changes and associations with symptoms can lead to the development of better therapies. Neurobiological explanations of the symptoms which are present in patients with AN have an effect on emotional reactions to the disease and influence the willingness to change; however, it is still difficult to translate brain findings into therapeutic strategies that improve symptoms, since brain research is still a relatively unexplored field. A consequence of the findings could be to recommend that therapy should focus on teaching AN patients more effective strategies to recognize and cope with fear, especially concerning bodily issues. Therapies which address the fear and anxiety of the patients, such as cognitive behavioral therapy could be used [39]. Mirror therapy could also be helpful to develop a more comprehensive appraisal concerning the distorted body image [41]. Since patients with AN have difficulties in downregulation of emotions, brain activation alterations in the processing of emotional stimuli in AN patients indicates that reappraisal with detachment strategy (low amygdala activation and high prefrontal activation associated with cognitive control) might be effective in therapy [18].
